# A Few Brief Remarks as Regards the Psychosis of Alcohol and Morphine

**Published:** 1914-02

**Authors:** J. Cheston King

**Affiliations:** Atlanta, Ga.


### A FEW BRIEF REMARKS AS REGARDS THE PSYCHOSIS OF ALCOHOL AND MORPHINE.

By J. Cheston King, A. B., M. D., Atlanta, Ga.

I am going to discuss in this paper first, the phychosis of morphinism, which is a most delicate subject with the medical profession. An eminent physician once said: "You might as well ask me to cure the habit of lying and stealing as that of drunkenness or that of opium." The moral obliquity of mankind does not come within the range of "materia medica." Is not this feeling a stigma on the medical profession, for the morphine habitues are not the willing slaves, so generally supposed, but in the largest per cent are the victims of medical carelessness or ignorance.

According to Pouchet, 40 per cent of all the morphinists are physicians, the wives of physicians and, also, druggists, dentists and chemists. In fact, the Medical Gazette de Paris states that 20 per cent of mortality amongst medical men was due to morphino-mania. Say, that even as low as 10 per cent of the medical profession are drug addicts, how appalling the situation.

Statistics show we have four millions of American people slaves to this drug. If the drug disease has been classed as incurable, then death is a blessing as compared with a lost individuality due to a mind gone wrong.

Since 1856 when Bertrand of Germany used the first injection of morphine, it has had a frightful and devastating career. Dr. Burnett in his rigorous and picturesque style has well said: "It extinguishes the patient's mental lights and thrusts him into a tophetic crucible, that would make hell a paradise and the accepted purgatory without sandles an oasis in Sahara. The patient lapses into Hades and expectorates Lucifer fumes, while the phosphorescent glow of his burning brain illuminates the strand where the fire boats put to sea."

The statement we have often heard made by eminent physicians that "once a drug addict, always a drug addict"—is not in accordance with latest research work, showing that the use of morphine, heroin, codine, laudanum or opium, produces a diseased condition of the nervous system, the stichochrome nerve cells are irregular, the chromatic bodies are enlarged, the cells of the cortex and medulla are extensively altered, showing clefts of the cell bodies, which shows that there is a short circuiting, fusing and burning out of the brain gangliomic cells. So we have to deal with a toxic neurasthenia, a disease of overstimulation, and the special indications are for cordial tonics, for a flagging heart deprived of its accustomed stimulation. The result of over stimulation is to produce metabolic perversion, and the fatigue of the nerve centers causes the formation of acids, which keeps up a condition of nervous exhaustion by chemical changes. In all cases of morphinism, there is found an excess of acid, and fatigue whether physical or mental, gives rise to formation of acids, and that stimulation consists of abnormal elicitation of energy, leaving afterwards reactive depression.

The withdrawal method which is generally accepted as best and safest, is accompanied by hyperacidity of the stomach, and to combat this acidity, the use of alkalines is indicated. Guimbail recommends the use of these alkalines in the form of vichy water. Bicarbonate also acts upon stasis and it is moreover a powerful tonic of the heart and circulation, at a time when it is necessary to act upon vaso-dilation, which caused as it is by general acidity is helped by the tonic action of bicarbonate. It is generally accepted now by French writers in modern textbooks, that heart tonics and vichy water are indicated from a therapeutic standpoint.

Crothers, who fitly represents American Medicine, says: "Great stress is laid upon the use during the entire treatment of preparations of soda to prevent or neutralize the acidity of the stomach or bowels. This is very essential in all methods of treatment."

It is maintained by some writers, and justly so I think; that morphia in the organism is changed by oxidation into what is called oxydemorphine and it is the presence of this in the blood and not the want of morphine that is the cause of the craving. Reasoning from this analysis, we should adopt a treatment that would comprise the promotion of "elementary discharges."

The morphinist is a man of double personality; after the injection he is in good humor, conciliating, capable of labor; after the cessation of its action, he is restless, repulsive, unable to concentrate himself and dull. A new injection reanimates him.

The most prominent pathological mental system which is brought forth by the abuse of morphine is the perversion of the moral feelings. Faith and belief and honor have become subordinate in his consideration. Loss of intelligence and energy follow.

The physical symptoms of chronic morphinism manifest themselves in general emaciation, insomnia, onstinate intentinal obstruction, trembling of the tongue and hands, weakness and ataxia of the arms and legs, contracted pupils with red conjunctiva. Accompanying these is loss of appetite, the tongue is rough and dry, the teeth are carious and fall out, the skin is sallow.

As stated above in this paper, in the treatment of gradual withdrawal, there will be found a hypersecretion of hydrochloric acid in the stomach, which may be shown by the aid of the stomach tube, hence pumping out the stomach and administering alkalies is indicated. There is one prophylactic measure of great importance, in attention to which is often the cause of failure, and which should be prescribed as a preliminary precaution in every morphia suppression. This is a thorough examination of the mouth. Morphia has always an injurious action on the teeth, causing caries and periaslitis. If the teeth have not been attended to, neuralgia will set up and be often the cause of relapse.

The main factors intended to be brought out in this paper are: first, that the use of opium or its derivatives produce a diseased condition of the nervous system, which is amenable to treatment and the condition of this patient should be studied as is the case with any other disease we come in contact with; second, when a physician prescribes this drug beyond actual medical necessity, he is warping the mentality, vitality and morals of the manhood and womanhood that go to make up the fibric of our great nation. Many nevelists, who with conspicious ignorance as regards the medical bearings of the subject, have written about the interaction of mind and body and arrived at some very dangerous conclusions.

I am free to confess that when Christian Scientists and Apostles of "new thought," shall have banished sin from the world, then will infectious disease be no more, specific tumors of the brain and aneurisms, now requiring iodide, will be cured by Eddist ejaculations, then will our honored profession, whose waves have touched all the shores of human thought; within which are all the tides and currents and pulses; upon which lay all the lights and shadows and over which brood all the calms and sweep all the storms and tempests, of which the soul is capable, meekly bow in submission to credensive induction, but that time will never be, for our work is builded on scientific lines, theirs is based upon credulity of dupes and false deductions are accepted as real causes.

Hewell Park Sanitorum,
Atlanta, Ga.

